# Genomic Variability in the Survival Motor Neuron Genes (*SMN1* and *SMN2*): Implications for Spinal Muscular Atrophy Phenotype and Therapeutics Development

**DOI:** 10.3390/ijms22157896

**Published:** 2021-07-23

**Authors:** Matthew E. R. Butchbach

**Affiliations:** 1 Center for Applied Clinical Genomics, Nemours Children’s Health Delaware, Wilmington, DE 19803, USA; Matthew.Butchbach@nemours.org; 2 Center for Pediatric Research, Nemours Children’s Health Delaware, Wilmington, DE 19803, USA; 3 Department of Biological Sciences, University of Delaware, Newark, DE 19716, USA; 4 Department of Pediatrics, Thomas Jefferson University, Philadelphia, PA 19107, USA

**Keywords:** spinal muscular atrophy, copy number variation, *SMN1*, *SMN2*, modifier gene, precision medicine, therapeutics, gene conversion, hybrid gene

## Abstract

Spinal muscular atrophy (SMA) is a leading genetic cause of infant death worldwide that is characterized by loss of spinal motor neurons leading to muscle weakness and atrophy. SMA results from the loss of *survival motor neuron 1* (*SMN1*) gene but retention of its paralog *SMN2*. The copy numbers of *SMN1* and *SMN2* are variable within the human population with *SMN2* copy number inversely correlating with SMA severity. Current therapeutic options for SMA focus on increasing *SMN2* expression and alternative splicing so as to increase the amount of SMN protein. Recent work has demonstrated that not all *SMN2*, or *SMN1,* genes are equivalent and there is a high degree of genomic heterogeneity with respect to the *SMN* genes. Because SMA is now an actionable disease with *SMN2* being the primary target, it is imperative to have a comprehensive understanding of this genomic heterogeneity with respect to hybrid *SMN1*–*SMN2* genes generated by gene conversion events as well as partial deletions of the *SMN* genes. This review will describe this genetic heterogeneity in SMA and its impact on disease phenotype as well as therapeutic efficacy.

## 1. Introduction

Proximal spinal muscular atrophy (SMA) is a leading genetic cause of infant death worldwide. SMA is an early-onset disease that is characterized by the loss of α-motor neurons in the anterior horn of the spinal cord, i.e., lower motor neurons [1,2]. The incidence of SMA is 1 in 6000–10,000 live births [3,4,5]. SMA has a carrier frequency of 1:25–50 in most populations [5,6,7,8], although it is lower for some ethnic groups [9,10,11,12]. SMA results from the loss of α-motor neurons in the ventral spinal cord, leading to denervation and muscle weakness, with proximally innervated muscles being preferentially targeted. Following onset of symptoms, the denervation is progressive over time, as shown in SMA patients using motor unit number estimation (MUNE) and maximum compound muscle action potential amplitude (CMAP) analysis [13].

Based on age of onset and severity of the disease, SMA can be classified into five distinct phenotypes [14,15]. Type 0 SMA infants present with very severe hypotonia and require respiratory support from birth. These SMA infants usually do not survive beyond 6 months. Type I SMA (Online Inheritance in Man (OMIM) database #253300) patients have an age of onset before 6 months and they present with limb weakness due to hypotonia and the inability to sit independently. Abnormal respiratory patterns have been observed in type I SMA infants due to weakness in the intercostal muscles but not the diaphragm. These patients typically have shortened lifespans. Type II SMA (OMIM #253500) patients have an age of onset before 18 months. They are poor crawlers and weak sitters; most of these patients can rarely stand and only with support. Their legs are generally weaker than their arms. These patients generally have a life expectancy into adulthood due to improvements in the standards of care. Type III SMA (OMIM #253400) patients have an age of onset greater than 18 months. These patients are able to walk with difficulty (waddling gait) and the legs are weaker than the arms. Type III SMA individuals generally live for a normal lifespan but some of them may require mobility support as the disease progress. Adult-onset (type IV) SMA (OMIM #271150) patients typically exhibit a slowly progressive limb weakness but the disease course is fairly benign.

While spinal motor neurons are the primary cell type affected in SMA, other types of cells aside from the motor neurons may also be affected by SMA [16,17]. For example, there are immature myoblasts present within muscles of SMA patients [18] and type I SMA patients tend to have smaller myotubes [19]. In addition to motor neuron degeneration, axonal degeneration of sensory neurons has also been observed in patients with severe SMA but not in milder forms of the disease [20,21]. Imaging and electrophysiology studies have shown degeneration of the thalamic nuclei within the cerebrum of type I SMA patients [22,23]. Type I SMA patients also manifest cardiac abnormalities including bradycardia and septal defects [24], while heart abnormalities are not observed in milder, types II and III SMA patients [25]. Distal digital necrosis of the blood vesssels occurs in type I SMA patients [24,26]. Type I SMA patients also show an abnormal increase in pancreatic islet α cells, leading to abnormal glucose levels in some patients [27]. Other metabolic manifestations observed in SMA include abnormalities in fatty acid metabolism in SMA patients [28,29,30] and elevated serum leptin levels [31]. The multisystem nature of SMA tends to be more clinically prominent in more severe forms of the disease. It is unclear at present if these systemic clinical manifestations are a consequence of motor neuron dysfunction but it is important, nevertheless, to consider these systemic clinical manifestations in the care for and treatment of SMA patients.

## 2. Genetics and Biology of SMA

**Figure 1:**
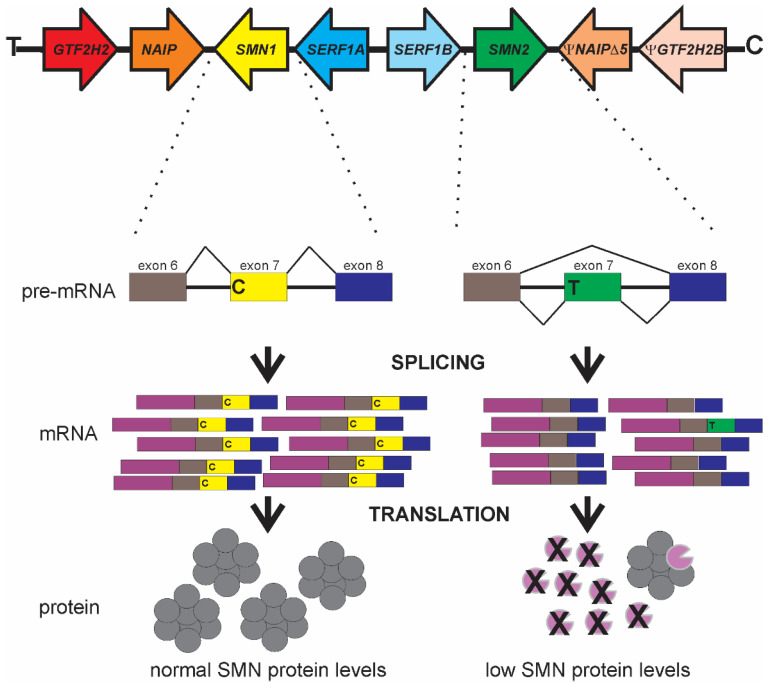
Genomic organization of the SMA-associated segmental duplication at chromosome 5q13 and the functional differences between *SMN1* and *SMN2* with respect to *SMN* gene regulation. Adapted from [42,43].

The SMA gene locus [32] maps to the 5q13 region of chromosome 5 (reviewed in [33]). Within this region there lies a 500 kilobase (kb) inverted segmental duplication that is unique to human lineages [34,35,36]. There are four genes within this segmental duplication region (Figure 1): *SMN* (survival motor neuron; [37]), *NAIP* (neuronal apoptosis inhibitor protein; [38]), *GTF2H2A* (general transcription factor IIH, p44; [39,40]) and *SERF1A* (small EDRK-rich factor 1A, *H4F5A*; [41]). These duplicated genes are either identical to their partner gene (*SERF1B*), differ by a small number of nucleotides but still produce functional genes (*SMN2*) or are pseudogenes (*ΨGTF2H2B* and *ΨNAIPΔ5*).

In more than 95% of cases, proximal SMA results from the loss of *SMN1* but retention of *SMN2*, regardless of clinical severity [37]. In more severe types of SMA, other genes within this region of segmental duplication (such as *NAIP*, *GTF2H2A* and *SERF1A*) may also be lost, but not always [39,40,44,45,46,47]. Intragenic mutations in *SMN1* [37,48] account for the remaining 5% of SMA cases (see Section 8) providing additional evidence to support *SMN1* as the gene responsible for SMA. In SMA patient-derived cell lines as well as in patient tissues, SMN protein levels are inversely correlated with disease severity [49,50,51,52,53,54,55,56]. SMN is a ubiquitously expressed protein that is required for the assembly of diverse ribonucleoprotein (RNP) complexes, including small nuclear RNPs (snRNPs) required for spliceosome assembly and messenger RNPs (mRNPs) needed for transport of mRNAs along axons [48,57,58]. While it is well established that SMN is required for RNP assembly, it still remains to be resolved which types of RNPs are affected in SMA and how motor neurons are preferentially affected. 

The major functional difference between these two *SMN* genes is a C-to-T transition in exon 7 (*SMN2 c.850C>T*) [59,60]. While translationally silent, this position on exon 7 is in the middle of an exonic splicing enhancer (ESE) sequence important for the inclusion of exon 7 in SMN transcripts (Figure 1). This ESE is disrupted in *SMN2*, thereby causing the exclusion of exon 7 (SMNΔ7) from the majority (~90%) of *SMN2*-derived mRNAs. The resultant SMNΔ7 protein is unstable and is unable to associate with itself [61,62,63]. Some *SMN2* mRNAs contain exon 7, depending on cell type, and can produce some full-length, functional SMN proteins. The SMNΔ7 protein is still partially functional since transgenic overexpression of SMNΔ7 in severe SMA mice partially ameliorates their phenotype [64].

## 3. *SMN2* as a Disease Modifier for SMA

The number of *SMN2* copies in the human genome varies between 0 and 8. Numerous studies have demonstrated an inverse relationship between *SMN2* copy number and disease severity in SMA [13,37,49,50,54,65,66,67,68,69,70,71,72,73,74,75,76,77,78,79,80,81,82,83,84,85,86,87,88,89,90,91,92,93,94]. Patients with milder forms of SMA have higher *SMN2* copy numbers than severe SMA patients. SMN2 copy number is being used as a prognostic tool to guide therapeutic strategies and care plans for SMA patients across the spectrum of phenotype severity [95,96]. The variability in *SMN2* copy number within the SMA patient population and its relationship to disease severity makes it an ideal target for therapeutics development.

Animal models such as zebrafish, fruit flies and mice have a single *Smn* gene which is orthologous to *SMN1* [97,98]. Loss of *Smn* in mice (*mSmn*) leads to embryonic lethality or cell type-specific death, if using conditional gene knockout approaches [99,100,101,102]. Transgenic insertion of *SMN2* rescues the embryonic lethality observed in *mSmn* nullizygous mice [103,104,105]. While two copies of *SMN2* rescues embryonic lethality in *mSmn*-deficient mice, these mice develop a very severe SMA phenotype and die within 8 days after birth [103,104]. Those *mSmn*-deficient mice with 3–4 *SMN2* copies exhibit a milder SMA phenotype than the two-copy *SMN2* SMA mice [104,105]. If the *SMN2* copy number is high (i.e., eight), then the resultant *mSmn*-deficient mice exhibit no signs of SMA and are phenotypically normal [103]. Introduction of *SMN2* onto a *Smn* nullizygous background in zebrafish also rescues embryonic lethality in this animal model [106,107]. *SMN2* CNV, therefore, is a major modifier of disease severity in SMA. 

## 4. Measurement of *SMN1* and *SMN2* CNV

Because *SMN2* copy number influences disease severity in SMA, there is prognostic value in accurate measurement of *SMN2* copy number from patients being evaluated for SMA. Molecular diagnosis of SMA—i.e., loss of *SMN1*—has historically been made using a polymerase chain reaction (PCR)-based assay followed by digestion of the PCR product with specific restriction endonucleases (PCR-RFLP) [37,75]. Different types of genotyping assays—including radioactive PCR [49,65], fluorescent PCR [79], quantitative (real-time) PCR (qPCR) [76,77,78], competitive PCR/primer extension [80], denaturing high-performance liquid chromatography [81], multiplex ligation-dependent probe amplification (MLPA) [82,83,84,85,86,108], quantitative capillary electrophoresis fragment analysis [87], short-amplicon melt profiling [88], fluorescent multiplex PCR/capillary electrophoresis [89,90] and universal fluorescent triprobe ligation [91]—have since been developed to quantify *SMN2* copy number in DNA samples from SMA patients. An important limitation of these established PCR-based copy number assays is the requirement for a parallel-run calibration curve to assign a necessary breakpoint that identifies placement of an ordinal *SMN2* value. Additionally, these techniques cannot easily distinguish unit differences in *SMN1* or *SMN2* when the copy number is greater than three [78,85,109]; however, recent refinements to MLPA assays can accurately measure four or five copies of *SMN1* or *SMN2* [110]. Digital PCR (dPCR) can accurately measure *SMN1* and *SMN2* over a large range of unit copies (0–6) without the need for an external calibration curve [70,93,111,112,113,114,115]. Next-generation sequencing approaches have recently been shown to be useful for SMA carrier detection [116,117,118,119,120] as well as for *SMN2* copy number measurements [120].

## 5. *SMN1* to *SMN2* Gene Conversions and Partial Deletions

**Figure 2:**
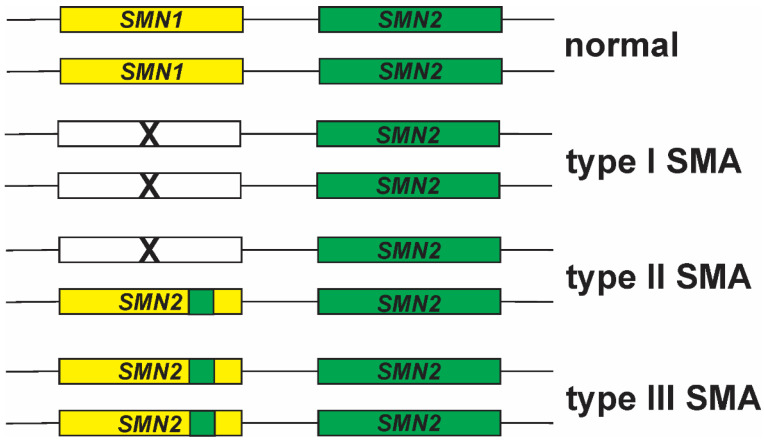
Relationship between *SMN1*–*SMN2* gene conversion and disease severity in SMA. Adapted from [134].

Gene conversion is one mechanism to account for increased *SMN2* copy number in the absence of *SMN1* in SMA [121]. In this scenario, the *SMN1* gene actually contains part of *SMN2*, in particular within exon 7 [46,122,123,124,125,126]. Gene conversion events between *SMN1* and *SMN2* have been observed by multiple groups using different approaches [89,93,122,123,124,125,127,128,129,130,131,132,133]. Gene conversion events may account for the inverse relationship between *SMN2* copy number and disease severity in SMA (Figure 2). Deletion of *SMN1* on both chromosomes is hypothesized to cause the more severe type I SMA. Milder forms of SMA result from conversion of *SMN1* to *SMN2* on one or both chromosomes (reviewed in [121]). Gene conversion events lead to the generation of hybrid *SMN* genes, i.e. some portions are *SMN1* while other sections of the gene are *SMN2*.

**Figure 3:**
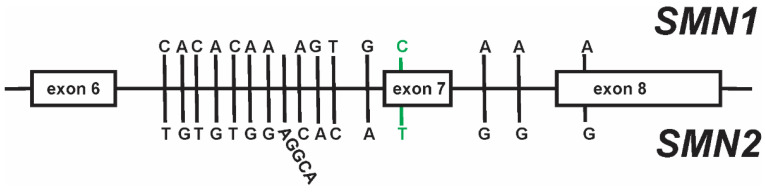
Paralogous sequence variants (PSVs) between *SMN1* and *SMN2*. The canonical PSV at exon 7 that functionally distinguishes *SMN1* from *SMN2* is highlighted in green.

Most gene conversion events occur at the canonical *c.840C>T* nucleotide difference at exon 7 [59,60]. There are, however, at least 15 other paralogous structural variants (PSVs) between *SMN1* and *SMN2* (Figure 3; [60,108,120,135,136]). Gene conversion events at exon 8 (*SMN2c.1155G>A*) as well as those within intron 6 (*SMN2c.835-44G>A*) and intron 7 (*SMN2c.888+100A>G* and *SMN2c.888+215A>G*) have been observed in SMA, as well as in control populations [127,129,133]. Some of these PSVs, such as *c.835-44G>A* and *c.888+100A>G*, can affect exon 7 inclusion in spliced *SMN* mRNAs [137,138]. Some hybrid *SMN2* genes produce greater amounts of SMN protein than expected and SMA patients harboring these hybrid genes have milder than expected clinical phenotypes. Further characterization of these gene conversion events will aid in the understanding of the functional consequence of these hybrid genes on *SMN* expression.

Even though the relationship between *SMN2* CN and disease severity is strong, there are exceptions to this inverse relationship. Some SMA patients displaying a type II or III clinical presentation only have two copies of *SMN2* as opposed to the predicted *SMN2* copy number for milder forms of SMA [139,140,141]. *SMN2* sequencing identified the presence of a rare single-nucleotide variant (*SMN2 c.859G>C*) in exon 7 [139,140,141]. This variant regulates the splicing of *SMN2* pre-mRNAs so that a greater proportion of *SMN2* transcripts contain exon 7. 

While most cases of SMA result from a complete loss of *SMN1*, partial deletions in *SMN1* have been identified in some samples from SMA patients—as well as in healthy controls—using PCR [142,143], microsatellite analysis [85,143], MLPA [83,85,108,110,127,142], whole-genome sequencing [120,135], long-range PCR [129] and dPCR [93]. Additionally, partial deletions have been observed in *SMN2*. The most common partial deletion of *SMN1* or *SMN2* encompasses exons 7 and 8 (*SMN1/2Δ78*) and is roughly 6.3 kb in length [120,135]. This partial deletion spans 6.3 kb of DNA, although it is possible that the sizes of these *SMN1/2Δ78* partial deletions may be variable. Whole-genome sequencing revealed the presence of a 1.9 kb partial deletion in a single sample that spans exon 7 and part of the flanking intronic regions [120]. In addition to *SMN1/2Δ78*, partial deletions within the *SMN* genes have been observed in other regions of the SMN genes, including losses of exons 5 and 6 (*SMN1Δ56*) [144], of exons 2a through 5 (*SMN1Δ2a5*) [145] and of exons 1 through 6 (*SMNΔ16*) [85,143]. We and others [93,142] have detected partial deletions of exon 8 in *SMN1* (*SMN1Δ8*). Even though this exon is downstream of the protein-encoding region of *SMN1* mRNA, it may affect *SMN1* mRNA stability as well as post-transcriptional gene regulation. 

The reason for specific breakpoints to be favored in partial deletion of *SMN1* is currently not known. There are numerous intrachromosomal repeats within human chromosome 5 [34], including within the SMA gene locus. Alu repeat elements are primate-specific, 300-base segments of repetitive DNA found throughout the human genome [146]. Within the *SMN* genes, there are numerous *Alu* repeat elements of different types [147]. Some of these *Alu* repeat elements may cause partial deletions of *SMN1* (or *SMN2*) by nonallelic homologous recombination [148]. The most common partial deletion (6.3 kb) of *SMN1/2*, *SMN1/2Δ78*, is flanked by *Alu* repeat elements [135]. Other partial deletions of *SMN1*—such as *SMN1Δ56* [144], *SMN1Δ2a5* [145] and *SMN1Δ16* [85,143]—are also flanked by *Alu* repeat elements. *Alu*/*Alu*-mediated rearrangements, therefore, may account for these partial deletions within *SMN1*.

## 6. *SMN2* Copy Number and Therapeutic Efficacy

The Food and Drug Administration has approved three therapeutic agents for SMA patients: nusinersen (Spinraza^TM^, Ionis Pharmaceuticals (Carlsbad, CA, USA) and Biogen (Cambridge, MA, USA) [149,150]), onasemnogene abeparvovec (Zolgensma^TM^, AveXis (Bannockburn, IL, USA) and Novartis (Basel, Switzerland) [151]) and risdiplam (Evrysdi^TM^, Genentech (South San Francisco, CA, USA) and Roche (Basel, Switzerland) [152]). Nusinersin and risdiplam act by increasing exon 7 inclusion in *SMN2* transcripts while onasemnogene abeparvovec replaces full-length *SMN* mRNA and protein. Since there is a strong relationship between *SMN2* copy number and disease severity, accurate and rapid measurements of *SMN2* copy number are often used to identify treatment options and regimens for children with SMA [96,153,154]. Accurate and rapid measurement of *SMN2* CN is particularly essential to guide decisions around timing and treatment choice for SMA infants identified by newborn screening. The impact of *SMN* hybrid genes and partial deletions on the responsiveness of these therapeutics has not yet been determined, but these atypical *SMN* genes are predicted to effect therapeutic efficacy, especially for nusinersin and risdiplam, as they are dependent on endogenous *SMN2*.

## 7. Intragenic Mutations in *SMN1* and SMA

As mentioned earlier, approximately 5% of all cases of SMA linked to 5q13 result from intragenic mutations within *SMN1* as opposed to the loss of *SMN1*. Table 1 provides a list of the currently known SMA-associated intragenic mutations in *SMN1*. The SMA-associated intragenic mutations located within the exons can be either missense, nonsense or frameshift mutations. Additionally, there are intragenic mutations within the intronic regions of *SMN1*, which can cause aberrant splicing of *SMN1* pre-mRNAs.

**Figure 4:**
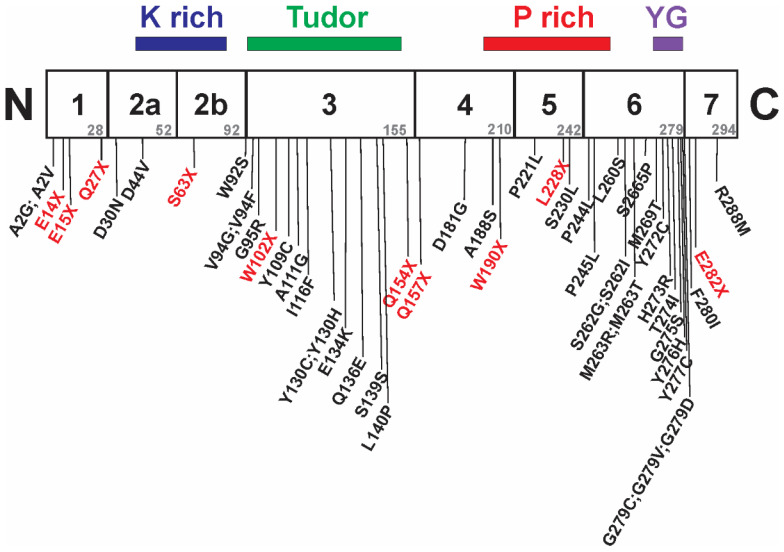
Location of SMA-associated intragenic missense and nonsense mutations within SMN1 relative to protein domain location. The mutated residues in red are nonsense mutations while those in black are missense mutations. Adapted from [48].

SMN is a highly conserved protein containing 294 amino acids (in humans) with multiple domains (Figure 4). There are three regions within *SMN1*—located within exons 2A (K rich domain), 3 (tudor domain) and 6 (YG box)—that are highly conserved evolutionarily [58]. Even though SMA mutations have been linked throughout *SMN1*, a greater proportion of SMA-associated intragenic point mutations are localized within these evolutionarily conserved regions (Figure 4). These conserved regions are required for self-oligomerization (YG box) as well as interactions with Sm proteins (tudor domain) and gemin-2 (K rich domain).

As mentioned previously, the loss of *Smn* in animal models is embryonically lethal and *SMN2* rescues this embryonic lethality but results in an SMA phenotype whose severity depends on *SMN2* copy number. Transgenic introduction of SMA intragenic missense mutations—specifically SMN1(A2G) [199], SMN1(A111G) [200], SMN1(D44V) [201], SMN1(T74I) [201] and SMN1(Q282A) [201]—into severe SMA mice (two copies of *SMN2* on an *mSmn* zulligygous background) improves the motor phenotype of severe SMA mice but does not completely ameliorate the SMA phenotype in these mice. These observations suggest that SMN genes harboring these point mutations are partially functional. 

On their own, none of these intragenic missense *SMN1* mutations can rescue the embryonic lethality of the loss of *Smn* in mice [199,200,201]. In zebrafish models for SMA where *zSmn* is knocked down with an antisense morpholino oligonucleotide [202], intragenic SMA missense mutations cannot rescue the motor axon deficits observed in these fish. These observations also support that these patient-derived point mutations are not fully functional. Interestingly, addition of both an N-terminal missense mutation and a C-terminal missense mutation can fully rescue the embryonic lethality caused by the loss of Smn in mice [203]. These intragenic complementation studies demonstrate that SMN must be oligomeric in order to function completely.

## 8. Silent Carriers and Compound Heterozygotes in SMA

**Figure 5:**
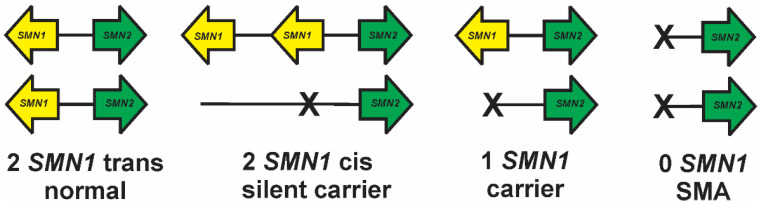
Allelic organizations of *SMN1* copies within normal (*SMN1*:1+1), silent carrier (*SMN1*:2+0), carrier (*SMN1*:1+0) and SMA (*SMN1*:0+0) patients.

Most parents of children with SMA are both carriers harboring one copy of *SMN1*. Interestingly, multiple independent studies have identified SMA carriers who have two copies of *SMN1* [65,204,205]. In fact, one study identified 4.3% of the SMA carrier parents within their cohort as having two copies of *SMN1* [205]. It is hypothesized that these so-called silent carriers have two copies of *SMN1* on one allele (i.e. the duplication allele) and zero copies of *SMN1* on the other allele (i.e., the deletion allele; Figure 5). In other words, the two copies of *SMN1* in a silent carrier have a *cis* allelic distribution (*SMN1*:2+0) as opposed to having two alleles each with a single copy of *SMN1* (the *trans* allelic distribution; *SMN1*:1+1), which would be phenotypically normal.

Luo et al. [206] recently identified two small variants within *SMN1*—*SMN1*g.27134T>G (also known as *SMN1*c.*3+80T>G) and *SMN1*g.27706_27707delAT (also known as *SMN1*c.*211_*212del)—that are tightly associated with silent carriers. The allelic frequencies of these variants were higher than expected in certain ethnic populations, such as the Ashkenazi Jewish and African American populations [206]. Another group identified an association between either of these variants and silent carriers in about 20% of their cohort, suggesting that there may be other variants in *SMN1* associated with silent carriers [128]. Alternatively, some instances of silent SMA carriers may not be linked with any structural variant within *SMN1*.

It is essential to develop diagnostic tools which can detect silent SMA carriers, as standard assays cannot readily distinguish *SMN1*:2+0 cases from *SMN1*:1+1. To facilitate the development of genotyping assays that can identify silent carriers, the Genetic Testing Reference Materials Coordination Program has identified a set of reference samples containing structural variants linked with silent carriers [207]. Recently, targeted next-generation and whole-genome sequencing approaches have identified silent carriers using the *SMN1*g.27134T>G single nucleotide polymorphism [117,120,208]. A quantitative PCR assay has also been developed to identify silent carriers using this variant [209]. It should be noted that most of these approaches will most likely not identify all silent SMA carriers, as not all of them are associated with these polymorphisms. Other approaches, including long-read PCR and sequencing, may provide additional ways to rapidly identify silent carriers.

Compound heterozygosity, wherein the SMA phenotype results from two different types of genetic event on each allele, can help explain discordant phenotypes within the families of SMA patients with differing phenotypic severities. One of the first cases of compound heterozygosity in SMA was identified by detailed analysis of haplotype markers [210]. In most cases, compound heterozygosity results from the deletion of one *SMN1* allele and an intragenic mutation within the other allele [133,155,166,179,192]. There have been cases where two different types of intragenic mutations, i.e., a frameshift mutation and a missense mutation, in *SMN1* occur on the same allele (cis) [87]. With new advances in molecular diagnostic tools, the genetics of complex cases of SMA can be resolved with relative ease.

## 9. Intrafamilial Variation in SMA Clinical Presentation

In some SMA families with more than one affected sibling, intrafamilial variability in clinical presentation has been observed [211,212]. In fact, recent analysis of a patient database curated by Cure SMA found 15.2% of SMA siblings to be discordant with respect to phenotypic severity [213]. These siblings have the same *SMN2* copy number but have differing clinical presentations [65,214,215,216,217]. This would suggest that there are additional genetic modifiers of SMA disease severity aside from *SMN2*. It is important to identify and characterize these novel modifiers for the development of new SMA biomarkers and targets for the development of therapeutic strategies for SMA [218].

*Plastin-3* (*PLS3*) was one of the first *SMN2*-independent modifier genes identified for SMA. On examining the transcriptomes of SMA families with discordant siblings, *PLS3* mRNA levels were found to be higher in females with milder SMA than those siblings with a more severe SMA clinical presentation [219,220,221,222,223,224]. The mechanism by which *PLS3* expression is altered in these discordant families remains to be resolved. Reduction in the expression of PLS3-interacting proteins *coronin 1C* (*CORO1C*) [225] and *calcineurin-like EF-hand protein 1* (*CHP1*) [226] improves neurite outgrowth in motor neurons from SMA model systems. A link between *CORO1C* and *CHP1* levels and disease severity, however, has yet to be shown in SMA patients. In some families, female siblings with a more severe SMA phenotype had higher *PLS3* mRNA levels than their more mildly affected siblings, suggesting that the protective effects of *PLS3* on SMA patients may be age- and sex-dependent or incompletely penetrant [222]. In the *Smn*^2B/−^ mouse model for SMA, SMA mice on a C57bl/6J genetic background lived, on average, over 30% longer than those mice on a FVB/N background [227]. Interestingly, *Pls3* levels were elevated in SMA mice on a C57bl/6J genetic background when compared against *Smn*^2B/−^ mice on a FVB/N genetic background. Ectopic overexpression of *PLS3* improved the survival and phenotype of SMA mice in some cases, but not all [228,229,230,231]. 

By using an approach combining linkage analysis with transcriptomics, *Neurocalcin-D* (*NCALD*) was found to be a potential modifier gene within a cohort of discordant SMA cases [232]. Targeted sequencing of the *NCALD* region identified a 17 bp deletion within the promoter region of this gene in these discordant SMA patients, which led to reduced levels of NCALD mRNA and protein.

Whole-exome sequencing of a discordant SMA family, where one sibling presented a milder SMA phenotype than the other sibling, even through they both had two copies of *SMN2*, identified point mutations in the *Tolloid-like 2* (*TLL2*) gene in the sibling with the milder phenotype [233]. TLL2 acts as an activator of myostatin (MSTN; growth differentiation factor 8), which inhibits skeletal muscle growth. The *TLL2* point mutations identified in the milder sibling are predicted to reduce MSTN activation. MSTN inhibitors (such as SRK-015) have shown therapeutic benefit in mouse models for SMA and are currently in clinical trials with SMA patients [234].

*Neuritin 1* (*NRN1*; cpg15) is an SMN-interacting protein present in neurons which promotes neurite outgrowth. Overexpression of *NRN1* in various animal models for SMA showed increased motor neurite outgrowth [235]. Yener et al. [223] recently showed elevated *NRN1* mRNA levels in a mildly affected sibling within a discordant family. The molecular basis for increased *NRN1* expression in this case remains to be resolved.

## 10. Conclusions

SMA results from the loss of *SMN1*, but retention of its paralog *SMN2* copy number can modulate disease severity in SMA. *SMN2* copy number is becoming an inclusion criterion for many clinical trials for SMA. Additionally, *SMN2* copy number can be used to help guide the type of care SMA patients will receive. Because of this relationship, *SMN2* is a primary target for the development of therapeutics for SMA [236,237]. Numerous targets of *SMN2* gene regulation—including promoter activation, increased inclusion of exon 7 and protein stabilization—are currently being developed to increase *SMN2* expression. Given the genomic heterogeneity of *SMN1* and *SMN2*, it will become very important to comprehensively assess the these genes in individual SMA patients, as some of them may harbor *SMN1*–*SMN2* hybrid genes or partial *SMN1/2* deletions that may affect the therapeutic efficacy of both current and future therapeutics.

## Figures and Tables

**Table 1:** Intragenic mutations in *SMN1* that have been identified in SMA patients. The nucleotide position for the mutation starts relative to the initiation codon for DNA (NM_022874.2) or amino acid for protein (NP_075012.1).

Type of Mutation	Mutation	Phenotype	Reference(s)
Nonsense mutations	p.E14X	I	[127,155]
	p.Q15X	I, II, III	[127,144,155,156]
	p.Q27X	I	[157]
	p.S63X	I	[157]
	p.W102X	II, III	[158,159]
	p.Q154X	III	[160]
	p.Q157X	II	[160,161]
	p.W190X	I	[162]
	p.L228X	I	[127,155,163,164]
	p.Q282X	I	[165]
	p.R288X	II	[166]
Frameshift mutations	c.-7-9del	III	[127]
	c.19delG	I	[164]
	c.22dupA	I, II, III	[127,155,163,164,167,168]
	c.48_55dupGGATTCCG	I	[87]
	c.56delT	II	[127,155,168]
	c.81+1dupG	II	[169,170]
	c.90_91insT	I, II	[144,162]
	c.98delT	I	[170]
	c.100delT	N/A	[171]
	c.109dupA	N/A	[158]
	c.124insT	I	[144]
	c.198_214del	N/A	[172]
	c.208_209ins4	III	[144]
	c.241-242in4	III	[144]
	c.286delG	I	[166]
	c.312dupA	III	[162]
	c.314_317dup	III	[172]
	c.401_402delAG	I	[164]
	c.411delT	I	[162]
	c.429_435del	I	[171]
	c.430del4	I, II, III	[173]
	c.431delC	I	[171]
	c.439_443del	I	[159,174]
	c.472del5	I	[174]
	c.509_510delGT	N/A	[158]
	c.524delC	N/A	[175]
	c.542delGT	II	[176,177]
	c.549delC	N/A	[178]
	c.551_552insA	I	[164]
	c.585dupT	I	[172]
	c.591delA	II	[144]
	c.627_628ins65	I	[161]
	c.722delC	I	[171]
	c.734_735insC	I	[160,175]
	c.735_736insA	N/A	[178]
	c.740dupC	N/A	[179]
	c.744delC	I	[127]
	c.770-780dup11	I	[158,160,162,172,175]
	c.773insC	III	[179]
	c.811_814dupGGCT	II	[127]
	c.813ins/dup11	I, II	[176,179,180]
	c.819_820insT	I	[157,181,182]
Missense mutations	p.A2G	II, III	[127,155,158,160,176]
	p.A2V	III	[129,157,182]
	p.D30N	II	[156]
	p.D44V	III	[156]
	p.W92S	I	[157,181,182,183]
	p.V94F	I	[184]
	p.V94G	II	[172]
	p.G95R	III	[156,178]
	p.Y109C	III	[127]
	p.A111G	I	[156]
	p.I116F	I	[160,175,185]
	p.Y130C	III	[158,186]
	p.Y130H	III	[186]
	p.E134K	I, II	[127,156,168,187]
	p.Q136E	I	[185]
	p.S139S	N/A	[158,159]
	p.L141P	I	[157]
	p.D181G	N/A	[188]
	p.A188S	I	[170]
	p.P221L	I	[87]
	p.S230L	II, III	[127,165,168,171]
	p.P244L	III	[171]
	p.P245L	III	[189]
	p.L260S	II	[172]
	p.S262G	III	[156]
	p.S262I	III	[65,158,190]
	p.M263R	I	[172]
	p.M263T	II	[162]
	p.S266P	II	[158]
	p.M269T	III	[160]
	p.Y272C	I, II, III	[144,156,162,164,170,172,189,191]
	p.H273R	II	[158]
	p.T274I	II, III	[71,144,170,190]
	p.G275S	III	[172]
	p.Y276H	I	[157,192]
	p.Y277C	II, III	[127,129,168,182]
	p.G279C	II, III	[178,193]
	p.G279V	I	[194]
	p.G279D	N/A	[143]
	p.F280I	N/A	[178]
	p.R288M	I, II	[168,195,196]
Splice-site mutations	c.*3+3A>T	I	[184]
	c.628-140A>G	N/A	[161]
	c.834+2T>G	I	[162]
	c.835-1G>A	III	[197]
	c.835-2A>G	I	[157,198]
	c.835-3C>A	I	[157]
	c.835-5T>G	I	[127]
	c.867+2T>G	I	[179]
	c.868-11del7	I	[37]
	c.888+3delAGAG	I	[37,172]
	c.922+3del4	I	[97]
	c.922+6T>G	III	[144]
